# Seven Years Trends in Prevalence of Transfusion-Transmissible Viral Infections in Yazd blood Transfusion Organization

**Published:** 2013-07-22

**Authors:** H Javadzadeh Shahshahani, M Vaziri, F Mansouri

**Affiliations:** 1 Assistant Professor of Blood Transfusion Research Center, High Institute for Research and Education in Transfusion Medicine, Tehran, Iran.; 2 Medical Doctor of Blood Transfusion Research Center, High Institute for Research and Education in Transfusion Medicine, Yazd, Iran.

**Keywords:** Prevalence, HIV, HCV, Blood Donors, Iran

## Abstract

**Background:**

Increasing blood supply safety is one of the most important goals of blood services in the world. In this study, we reviewed the prevalence rate and the trends of three main infections in whole blood donations and strategies for improving blood safety in Yazd blood transfusion center, Iran.

**Materials and Methods:**

In this cross sectional study, data on hepatitis B, C and HIV infection were extracted from Iranian Donor Database of blood donation from 2004 to 2010 in Yazd province. All donors with positive confirmatory test were included. The data was analyzed by SPSS software due to demographic factors.

**Results:**

The prevalence rate of hepatitis B, C and HIV infection decreased during these years (From 0.37%, 0.14% and 0 percent in 2004 to 0.14%, 0.05% and 0 in 2010, respectively). Both hepatitis B and C infections were significantly more in first-time blood donors with BSc or BA educational level. The prevalence rate of hepatitis B was significantly higher in donors with less than 20 year-old and female donors. The prevalence rate of hepatitis C was higherin30-39 age group (P-value= 0.014).

**Conclusion:**

The results showed that the strategies used for improving blood safety were efficient. Increasing public knowledge on blood-borne infections and their routes of transmission, importance of donating blood only by healthy donors are necessary to have a safe blood supply in future.

## Introduction

Prevalence evaluation of blood borne infections in blood donors is useful to assess blood safety. Blood transfusion centers are an important part of National Health Care system and their goal is to maintain adequate healthy and easily available blood components. 

Several methods have been used to increase blood safety: society and blood donors' educational programs, adoption of better donor selection criteria, improvement of more sensitive and specific laboratory tests to detect Hepatitis B virus (HBV), Hepatitis C Virus (HCV) and Human Immunodeficiency Virus (HIV) in donated blood (1-5).

The prevalence of blood borne infections has been evaluated in many world regions and resulted in different rates. These differences are because of variation in the prevalence rate in general population, types of donation (voluntary or replacement), screening methods and laboratory tests in different societies (6-8).

In Iran, changes in prevalence of blood borne infections had been previously studied (9-12). In this study we evaluated the trends of HBV, HCV and HIV infections in blood donors in correlation with demographic data and blood donor's status (first-time, lapsed and regular donors) from 2004 to 2010 in Yazd province. 

## Materials and Methods

In this descriptive and analytical study, retrospective review of donor’s record covering the period during 2004 - 2010 at Yazd blood transfusion center was carried out. Data on donation numbers, donation status, demographic characteristics and screening and confirmatory test results of HBV, HCV and HIV were obtained from the Iranian Blood Services National Epidemiology Donor Database. 

The donations were all screened for HBs-Ag, anti-HIV, HIV antigen/antibody (from 2005), anti-HCV according to standard operating procedures by approved commercial kits (10). Confirmatory tests included the monoclonal neutralization assay for HBs-Ag, the third-generation recombinant immunoblot assay (RIBA 3.0) for HCV and HIV-I/II Western blot (WB) assay for HIV infection (10).

Donors between 18 and 65 years were selected through vital sign examinations, anemia screening and medical history and risk behavior interviews, which are routinely performed by trained physicians. A first-time blood donor was identified as a donor who donated for the first time. A regular donor was defined as a donor who donated more than once during one year and a lapsed donor was any donor who had a history of previous donation, but the interval between two donations was more than one year. Before blood donation, a consent form was signed by all blood donors.

**Statistical Analysis**

Frequency of HBs-Ag, HIV antigen/antibody, anti-HCV antibody per 100,000 donations and 95% confidence interval (CI) were calculated using a binomial distribution. The prevalence rate of infections was calculated in each demographic group, and compared using chi-square test and considered significant if p-value was less than 0.05.

## Results

In total, according to the database records, there were 254760 allogenic whole blood donations during 2004 to 2010 at Yazd blood transfusion service. Among whom ninety six percent (96%) were male and four percent were female. 33% were first time, 26% were lapsed and 41% were regular donors. 

**Fig 1:**
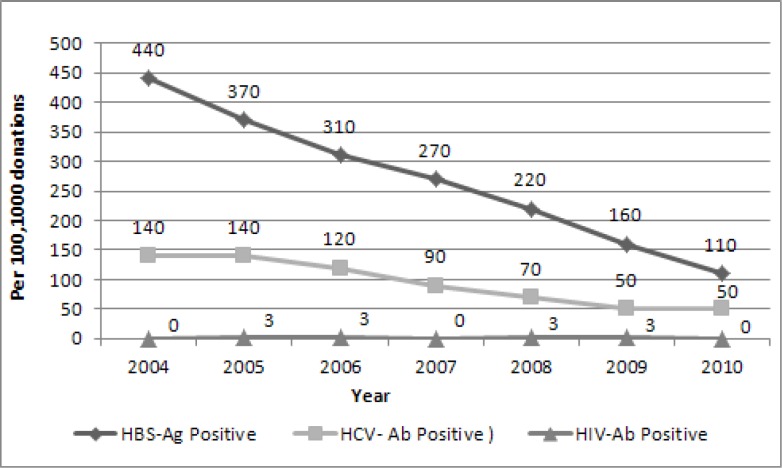
Trends of HBS-Ag. HCV-Ab and HIV-Ab from 2004 to 2010 in Yazd blood transfusion center

Among all donors there were 4, 239 and 667 confirmed positive tests for HIV, HCV and HBV infections, respectively. Temporal trends of these three blood borne diseases during 2004 to 2010 are shown in Fig 1.

**Fig 2:**
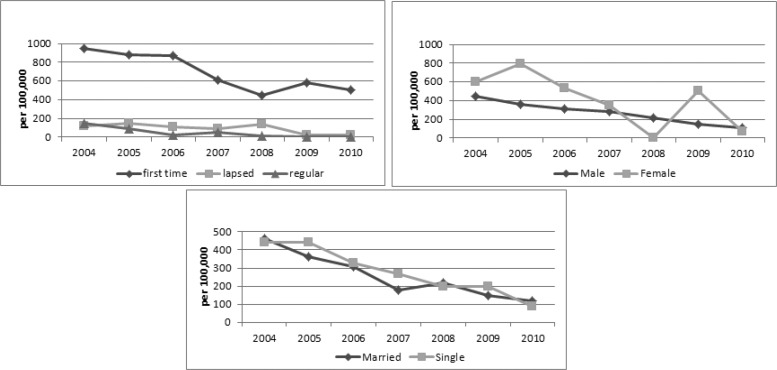
Trends of HBS-Ag positive rate according to gender, marital status and donors' status

Table I shows prevalence of hepatitis B and hepatitis C infections in Yazd blood transfusion donors according to their demographic characteristics from 2004 to 2010.256 per 10^5^ donations of male blood donors and 402 per 10^5^ donations of female blood donors were HBs-Ag positive (P-value= 0.002). The rate of first-time donation was significantly more in females than males (P-value< 0.001). 50% of female donors and 32% of male donors were first-time. Among 667 confirmed HBs-Ag positive donations, 568 (690 per10^5^ donations) were first time, 59 (90 per10^5^ donations) were lapsed and 40 (40 per10^5^ donations) were regular donors (P-value< 0.001).The trend of HBs-Ag positive rate was decreasing due to donor's status (Fig 2). 

**Fig 3:**
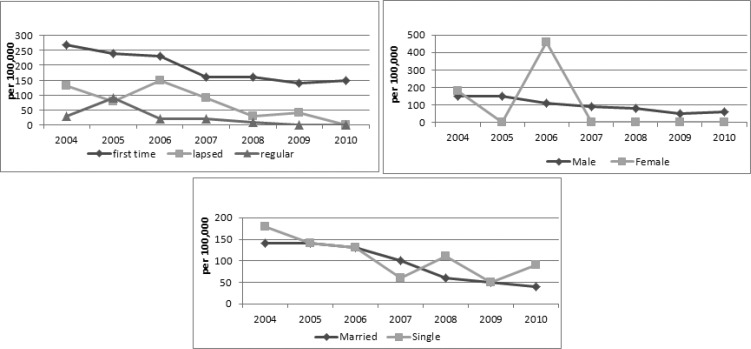
Trends of HCV-Ab positive rate according to gender, marital status and donors' status

During 2004 to 2010, 239 donors had confirmed positive test results for HCV-Ab. The trend of HCV-Ab positive test was decreasing (Fig 1).According to age, the 30-39 age group was the most prevalent group for HCV-Ab (P-value= 0.014). HCV-Ab positive donations were significantly more prevalent in BSc and BA and high school groups (P-value< 0.001) (Table I). The trend of HCV-Ab positive rate due to blood donor's status was decreasing (Fig 3). HCV-Ab prevalence rate was 205, 72 and 21 per10^5^ donations of first time, lapsed and regular donors, respectively (P-value< 0.001).

There were four people, who were positive for HIV-Ab in 2005, 2006, 2008 and 2009. The prevalence rate was three per 10^5^ donations among each of these years. All these donors were married male and first-time donors. Except for an illiterate HIV positive donor in 2006, all of others were high school graduated.

**Table I:** Positive rates of HBs-Ag and HCV-Ab tests in blood donations, grouped in different demographic characteristics in Yazd blood transfusion center, 2004-2010

	**HBs-Ag**				**HCV-Ab**	
**Demographic factor**	**number of positive donations**	**Prevalence rate in 100,000 donations **	**95% CI per 100,000 **	**number of positive donations**	**Prevalence rate in 100,000 donations **	**95% CI per 100,000**
**Male**	626	256	240-281	230	96	83-108
**Female**	41	402	297-558	9	94	33-155
						
**First-time**	568	690	633-746	169	205	174-236
**Lapsed**	59	90	66-112	48	72	52-93
**Regular**	40	40	27-52	22	21	13-31
						
**Married**	516	264	242-287	180	92	79-106
**Single**	151	275	231-319	59	107	80-135
						
**<20 years old**	31	246	159-332	5	39	5-74
**20-29 years old**	132	139	115-163	61	64	48-80
**30-39 years old**	88	122	97-148	66	92	70-114
**40-49 years old**	84	170	133-206	25	50	31-70
**≥50 years old**	41	160	111-108	11	43	18-68
						
**Illiterate**	18	400	218-592	4	90	2-178
**Secondary school**	92	100	78-119	51	55	40-70
**High school**	428	510	462-558	158	188	159-218
**Diploma**	63	100	75-125	12	19	8-30
**BSc/BA**	63	1250	940-1551	12	237	103-371
**MSc/MA and above**	3	60	0-134	2	42	0-100
**Total**	667	267	247-287	239	96	83-108

## Discussion

The study results showed that transfusion-transmissible infections (TTIs) had a decreasing trend during 2004 to 2010 (Fig 1). This trend is more dramatic for HBs-Ag (from 440 per 105 in 2004 to ll0 per 105 donations in 2010). The frequency of HCV-Ab has been decreased from 140 per 105 donations in 2004 to 50 per 105 donations in 2010. In Yazd blood transfusion center, some strategies that are used to increase blood supply safety are: The act of donor counseling and donor selection performed by trained physicians, Regular counseling educational workshops are provided for them , Integrated software is used for donor registry and donor selection in all donor centers including mobile teams in the province, Surveillance of TTI markers in mobile teams’ donations is performed, Those regions with high prevalence of positive markers are omitted, Increasing in regular blood donor rates by persuading healthy first time and lapsed donors to donate blood regularly at least two times a year, Detecting people who would like to donate blood only to be tested for TTIs and referring them to health care centers where they would be tested for free, Implementing confidential unit exclusion program in blood centers and more awareness of donors about transmission routes and self – avoidance of blood donation in the case of high–risk behaviors.

Lower frequency of viral infections in regular donors than lapsed and first time donors could be due to more awareness in this group or elimination of donors with initial reactive screening tests. The decreasing slope for HCV- Ab was slower than HBs-Ag. The HBs-Ag prevalence rate in first – time donors has been increased in the last two years (2009-2010). Probably it is the result of increase in infection prevalence in general population. This is similar to studies performed in Kuwait, France and The USA (3, 4, and 13). In the study's period, the prevalence of HBs-Ag was significantly more in females (P-value= 0.002). Analysis showed that first–time donations were significantly more in females and it could be cause of higher prevalence of HBs-Ag in this group (P-value< 0.001). However, according to studies in the USA, France and Shiraz (Iran), the prevalence of both HBs-Ag and HCV-Ab was more in males than females (3, 4, 9).

In lapsed and regular donors, the rate of HBs-Ag was zero in the last two years that shows more accuracy in donor selection and more education of donors.

Due to age classification, below 20 year-old and between 40 to 49 year-old groups were the most prevalent groups for HBs-Ag (P-value< 0.001). In Kuwait, below 39 year-old, in France between 40 to 49 year-old and in Saudi Arabia above 50 year-old groups were more prone to hepatitis B (3, 13, and 14).

The prevalence rate of HCV-Ab due to donation status had decreasing trend during study’s period. There was a significant difference between HCV-Ab prevalence rate among regular, first – time and lapsed donors. The prevalence rate among first – time donors was three times higher than lapsed and 10.5 times higher than regular donors that is almost similar to Kuwait, the USA, France and Shiraz (Iran) (3,4,9,13).

The frequency of HCV-Ab among donors with different gender and marital status did not have any significant difference. In the USA and Shiraz (Iran), it was higher in males (4, 9). In Saudi Arabia and France, there were not any significant difference between males and females (3, 14).The 30-39 year-old group was the most prevalent group for HCV infection in our study. This result is similar to study of Amini et al but there were significant associations between increasing age and HCV in a study in Brazil (2, 12). The prevalence rate of HCV was higher in below 39-year-old group in Kuwait (13).

The prevalence rate of HIV in general population in Iran was 0.008 and 0.023 percent in years 1999 and 2007, respectively. Despite increase in prevalence rate of HIV infection in general population, the prevalence rate in whole blood donations did not increased during the study period. In this study, the prevalence rate of TTIs was less than neighbor countries such as Pakistan and Turkey (15-17).

## Conclusion

Totally, lower prevalence rate of TTIs in blood donors and its decreasing trends shows that the safety procedures performed at Yazd blood transfusion organization during these years were efficient. However, these rates could be more under control by using implementation of nucleic acid test (NAT) for detection of HBV, HCV and HIV in blood donations and education of society about routes and risk factors for transmission of TTIs, high-risk behaviors and the importance of blood donation by healthy donors.
